# Time and Its Value in the Practice of Dentistry
*Read before the Lebanon Dental Association, at Harrisburg, Pa.,


**Published:** 1904-10

**Authors:** W. H. Scholl

**Affiliations:** Reading, Pa.


### TIME AND ITS VALUE IN THE PRACTICE OF DENTISTRY.*

By W. H. Scholl, D. D. S., Reading, Pa.

When man is ushered into this world there is no guarantee issued at his birth as to how long he is to live here. If he had a knowledge of the hours, days, or years he is destined to remain upon earth, man's time could be divided and subdivided so that he could spend so many years in educating himself for his life-work in whatever field the trend of his ambition led him, and also set apart the number of years for the gratification of all his longings. But as there is no certainty of his stay here, man should remember Seneca's admonition, "Live as if every moment were to be thy last." If endowed with healthy organs, supplied with proper nutriment and the needed sunshine and exercise, there is no reason, barring accidents, why man should not live the allotted span of life, with confidence expect to reach a ripe old age, and divide his time accordingly.

Time is defined as being "the measure of the duration, whose parts are marked by the motions of the heavenly bodies, as a year, a month, a day, and by the artificial divisions or aggregates of these."

*Read before the Lebanon Dental Association, at Harrisburg, Pa.,

Fuller says, "Make use of time, as thou valuest eternity," and Franklin remarks, "Dost thou love life? Then waste not time, for time is the stuff that life is made of." To Thomas Jefferson is ascribed the wise utterance that "No person will have occasion to complain of the want of time who never loses any," and Mason says, "As every thread of gold is valuable, so is every minute of time." Fénelon remarks that "Time is given us that we may take care of eternity; and eternity will not be too long to regret the loss of our time if we misspend it." "Time, the cradle of hope, but the grave of ambition, is the stern corrector of fools, but the salutary counselor of the wise, bringing all they dread to the one and desert to the other." Such is the beautiful definition of time by Cotton.

Horace Mann seeks to impress the worth of time thus: "Lost yesterday, somewhere between sunrise and sunset, two golden hours, each set with sixty diamond minutes. No reward is offered for they are gone forever!" Undoubtedly, the versatile and immortal Franklin presents the value of time in its most impressive form when he claims it as his "silent partner." Listen again to this acute and ingenious author: "If time be of all things the most precious, wasting time must be the greatest prodigality, since lost time is never found again; and what we call time enough always proves little enough. Let us then be up and be doing, and doing to the purpose; so by diligence shall we do more with less perplexity."

But what I wish to emphasize on this occasion is the value of time as we measure it by appointments with our patrons. It has been said that time is the only capital possessed by the dentist. If this is true, we can readily understand its importance, for it is the use we make of time which establishes its worth. It is our bread-producing capital, and the seconds, minutes, and hours lost are past redemption. So, if it is not our entire capital, it is, certainly, the most vital part thereof, because of its ever-fleeting nature, for a moment lost is gone forever. Its significance is measured by its transitory character.

If time is the capital of the dentist, it is also the capital of the great majority of mankind, and even of those of ample means, for it is not the nature of man to live on what he has, nor is it recognized as a quality of good citizenship not to be a producer of some kind, nor should the world expect to realize something for nothing. The physician's time may be of more value when he is called in a case where the issue is one of life and death than is the dentist's; but in the majority of cases the physician, if need be, can shorten his calls, while usually the dentist cannot without sacrificing his efficiency or slighting his work.

In serious illness medical aid is summoned with all speed, and the physician calls daily as long as his services are needed—sometimes a little longer,—depending often on the length of the patient's purse. The credulity of man, it is said, is great, and if a physician has the persuasive faculty well developed and is unscrupulous, he can inoculate a susceptible mind with the fear of the most dire consequences if his services are not continued and his advice not heeded.

While death is not often threatened by a disturbance in the dental organs, nevertheless the sufferer rushes to the dentist, gets relief, swears that henceforth he will have his teeth attended to, engages time, returns home with the pain gone, and becomes oblivious to the engagement. The dentist waits patiently; the hour passes, but the sufferer has been so well cured that he has forgotten his appointment. If reminded of the fact by a gentle request in the form of a bill for services rendered, he waxes wroth, and threatens with dire vengeance the alleviator of his suffering. If I could collect the sums due me for time lost by unkept engagements in my forty years' practice, I might occasionally be able to play golf or go fishing.

The liability of patients to pay for time engaged but not used is beyond dispute, for the courts have so decided in many instances; but as the dentist is dependent on the good will of his patrons, it is seldom wise to go to law in order to recover.

Every patient should be given an engagement card with the date punched or marked and the penalty stated if the appointment is not kept. The question of fees has never been a subject of discussion in any one of the three dental societies to which I belong. It is questionable if any equitable schedule of fees could be formulated that every one, even in the same community, could adhere to, unless a sliding-scale were attached allowing much discretion on the part of the operator to accommodate his charges to the varying circumstances of his patients. It is greatly to the honor of these bodies that they have never argued this question, as it might be held to justify the community in the opinion that our organizations are on the same footing as societies whose chief concern is that of wages. I certainly do not desire to start a discussion on the subject today, but at the same time I see no reason why a local body could not have some understanding of what is a fair remuneration for our work. The tedious, nerve-exhausing labor a dentist is called upon to do, together with the expensive material, tools, and machinery he has to employ, including the comfortable rooms he has to maintain, should bring a fair return.

Dr. W. H. Atkinson said, "All difficult labor is necessarily capable of commanding higher compensation for the time spent." Some dentists cater for a rich patronage and can command higher fees. Then again some people measure the qualifications of a dentist by the style he lives in and the amount of his charges,—the higher the better. I have heard some dentists speak very contemptuously of others on account of the small fees they received. But big fees are not always a criterion as to qualification, for the worst quacks very often make the most money.

I acknowledge that I am unable to quote you a scheduled table of fees to go by. I could never encourage nor find any comfort in the method of getting all you can out of a patient on the "squeeze him for all he is worth" principle. Such conduct in a dentist is unprofessional and worthy only of swindlers and highwaymen.

That the expert and proficient dentist is able to command higher fees should be no cause for jealousy among the less expert. Rather it should be an incentive to gain the same ability. As a dentist is a human machine, subject to wear and tear, and can no more endure continuous work than his fellow-beings, even if he overtops them intellectually,—he must abide by the wise injunction to "rest from labor" at due intervals.

Some of us may think that the fees received here are entirely too low. This question was discussed by prominent dentists in New York City in 1863. Dr. J. S. Latimer (who, by the way, was the first man that produced a first-class barbed dental nerve-broach) stated that his charges were three dollars per hour and one dollar an hour for lost time. The fees in Philadelphia vary from three dollars to eighteen dollars per hour. The late I. H. McKellops, of St. Louis, is reported to have received fifty dollars per hour. Of the lamented William K. Atkinson—one of the best gold operators that ever filled a tooth—it was said that he died poor because he sought to establish a twenty-five-dollar-per-hour fee. This story is, however, emphatically denied by Dr. Mills.

To a sensitive man there is an unpleasant and sometimes a mortifying feature about asking high or exorbitant fees, as the following stories of a New York dentist will show:

A lieutenant of the navy was asked one hundred dollars for filling a tooth; the officer offered, in addition, his gold watch and chain, saying, "If you want to rob me take these also." In another instance, after putting the mouth of a Quaker in good condition, he requested $2,700 for it. The Quaker demurred very strenuously. The doctor said: "I have taken thee to my bosom and have befriended thee. I have put thy mouth into a healthy condition, so that thee can masticate and insalivate thy food, relieving thy stomach from overwork, thereby keeping it healthy, so that its function can be properly performed. I have befriended thee as a brother, and thee should not hesitate to pay the paltry sum I am asking thee." I do not begrudge them the fees who can command a rich clientele. Let them have all the honor that is due them. But the great majority of just as good and competent operators who are willing to serve the people at a rate corresponding with the means of their patrons are possibly deserving of as much praise and as high a rank—if not higher—than those catering only to the rich. The parted lips of the hundreds you meet everywhere display sufficient imperfections in their teeth for hundreds of additional dental co-laborers. There is room at the top for all who are willing to work up from the lowest round.

The young man who learns a trade receives a certain amount of pay and an increase every six months or year until he has served his apprenticeship, when he at once gets the ruling wages prevailing at that place. The student in dentistry must be eighteen years of age, twelve of which are necessary for the preliminary educational training required to make him an intelligent dental student, able to grasp the subjects daily presented for his study. Instead of earning money, as is the mechanic, he is spending it. While the mechanical apprentice is earning one thousand dollars, the dental student is spending at least two thousand dollars. This added to the one thousand dollars the mechanic is earning, equals three thousand dollars. Add to this sum the cost of instruments, etc., which at the lowest is five hundred dollars, and this will hardly include office furniture. So in the very start the new dentist has a debt of three thousand five hundred dollars to four thousand dollars. It is immaterial where the money came from,—it's the financial capital that the dentist must calculate on, while out of time, his "silent partner," the interest and his living expenses, etc., must be earned. It is poor policy to start with inferior tools and appliances, because time is the most expensive commodity around the dentist's office: therefore, any device, preparation, or tool that saves time saves money.

It is not the province of this paper to specify what fees a dentist should receive for his time and materials; undoubtedly he has to be satisfied with what his skill can command; but a good rule to go by is to increase your fees as the demand for your services increases.

Time has wrought wonders in the improvement of dentistry since my entrance into the profession. In those days a paying reputation was only gained by years of practice, and the most convincing arguments were good and lasting fillings or dentures. But in order to demonstrate the virtue of operative dentistry a good deal of work had to be done at a mere pittance. Thanks to progress, mistrust and superstition have given way to reason and common sense. Even the most illiterate recognize that no one is ugly who displays a fine, clean set of natural teeth however irregular. As the late Professor George T. Barker remarked in his strenuous style, "there never has been, nor can be made an artificial substitute—however excellent—that can equal a moderately sound natural set."

On the importance of time I have quoted the thoughts of men in many vocations of life and have endeavored to impress you with its value in relation to our daily routine of work. As most people have to sacrifice time in order to avail themselves of the services of the dentist, he should be promptly on hand when an engagement is due, for he cannot expect punctuality in others if himself unpunctual.

In reading this paper I have not been sparing of your time, and for your forbearance with that trespass extend my hearty thanks.

